# Heart-Brain axis: is microvascular dysfunction the link between stroke and Takotsubo syndrome?

**DOI:** 10.1007/s10554-025-03477-9

**Published:** 2025-08-01

**Authors:** Riccardo Cau, Michele Porcu, Jasjit S. Suri, Filippo Cademartiri, Mahmud Mossa-Basha, Luca Saba

**Affiliations:** 1 Department of Radiology, Azienda Ospedaliero-Universitaria (A.O.U.) di Cagliari, Polo di Monserrato s.s. 554 Monserrato, Cagliari, Italy; 2 University of Cagliari, Cagliari, Italy; 3 Department of ECE, Idaho State University, Pocatello, ID 83209 USA; 4 Department of CE, Graphics Era Deemed to be University, Dehradun, 248002 India; 5 University Center for Research & Development, Chandigarh University, Mohali, India; 6 Symbiosis Institute of Technology, Nagpur Campus, Symbiosis International (Deemed University), Pune, India; 7 Stroke Diagnostic and Monitoring Division AtheroPoint™, Roseville, CA 95661 USA; 8 IRCCS SYNLAB SDN, Naples, Italy; 9 Vascular Imaging Lab, Department of Radiology, University of Washington, Seattle, WA USA

**Keywords:** Microvascular dysfunction, Takotsubo syndrome, Stroke, Cerebrovascular diseases

## Abstract

Takotsubo syndrome (TS) is characterized by transient left ventricular dysfunction, often triggered by psychological or physiological stress. Increasing evidence highlights the critical role of the brain-heart axis in TS, with small blood vessels acting as central mediators. Recent data indicate a significant association between TS and cerebrovascular events, particularly ischemic stroke. Studies show that the incidence of stroke in TS patients is 1–2% per patient-year, which may occur during the acute phase or later due to ongoing autonomic and microvascular dysfunction. This underscores the shared mechanisms of microvascular impairment between the heart and brain in TS. Imaging techniques are essential for detecting microvascular abnormalities in both the heart and brain, providing valuable insights into the interconnected nature of microvascular dysfunction across the heart-brain axis. The aim of this review is to investigate the potential shared pathophysiological mechanisms that link TS and stroke, with a specific emphasis on microvascular dysfunction as a common factor. By examining the role of the heart-brain axis in both conditions and emphasizing the crucial role of advanced imaging techniques, this review seeks to clarify how microvascular abnormalities can simultaneously affect the cardiovascular and cerebrovascular systems. Additionally, it provides insights into the clinical implications of these findings, highlighting the importance of viewing TS and stroke as interconnected conditions within a shared pathological framework. Understanding these mechanisms may not only improve early detection but also pave the way for novel therapeutic strategies targeting both cardiac and cerebral microvascular health, potentially enhancing outcomes for TS patients.

## Introduction

Takotsubo syndrome (TS), also known as stress-induced cardiomyopathy, is an acute heart condition that closely mimics the clinical presentation of acute myocardial infarction [1–5]. Although TS was once considered a benign and self-limiting disorder, recent studies have challenged this view [6–9]. Several studies have reported a link between TS and cerebrovascular events, particularly ischemic stroke [10–13].

Data from the International Takotsubo (InterTAK) Registry, confirmed the coexistence of TS and stroke, raising the hypothesis that a common underlying mechanism might link these two conditions, rather than a simple cause-effect relationship [14].

Although the exact pathophysiological mechanisms connecting stroke and TS are still poorly understood, it is thought that the “Heart-Brain axis” plays a pivotal role in this connection [15–18]. The connection between TS and stroke may stem from shared mechanisms like endothelial injury, oxidative stress, and sympathetic overactivation. Excess catecholamines in TS impair vascular function and increase oxidative damage. This can disrupt both myocardial perfusion and the blood-brain barrier [19–21].

**Fig. 1:**
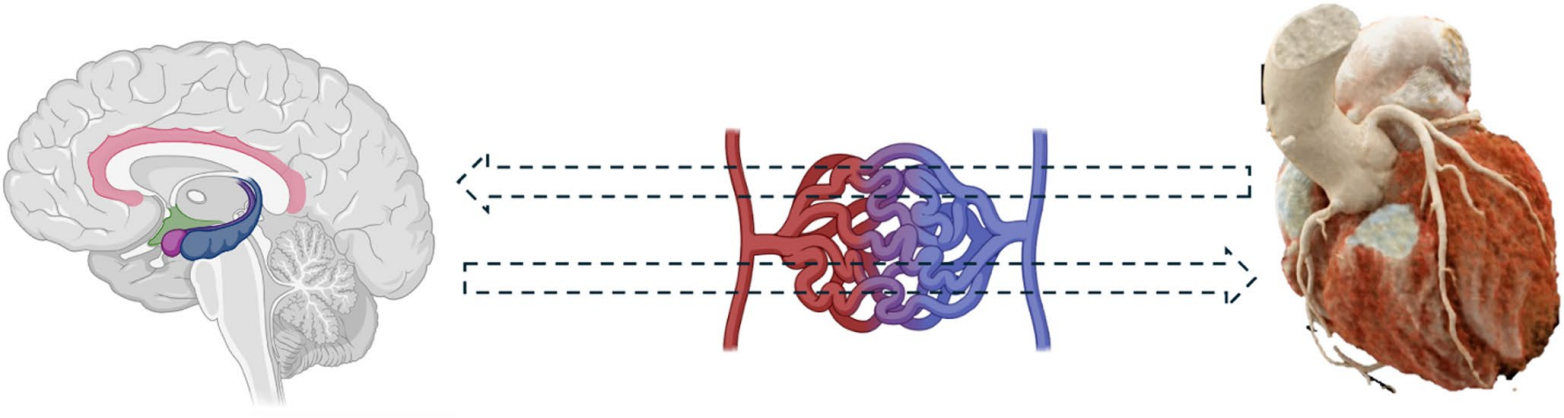
Graphical representation of the brain–heart axis in Takotsubo Syndrome (TS) and stroke, with a focus on microvascular dysfunction

Traditionally, stroke was viewed as either a trigger or complication of TS. However, this binary perspective is evolving. It is now recognized that the heart and brain share similar vascular features, and both may be affected by a common upstream abnormality [18]. Dysregulation of this shared vascular network may contribute to the pathophysiology of both conditions. Instead of TS and stroke causing each other, it is now proposed that both may be independently triggered by a common underlying factor, such as microvascular dysfunction. This impairment can affect small arteries and arterioles, leading to reduced blood flow to the heart, causing TS, or to the brain, resulting in stroke [22]. Figure 1.

Microvascular dysfunction in both the heart and brain can arise from several key etiologies, including ischemia, reperfusion injury, and endothelial stunning. These processes often converge on common final pathways, such as the production of reactive oxygen species and activation of inflammatory responses, which are frequently triggered by shared cardiovascular risk factors [23, 24].

Imaging modalities play a crucial role in evaluating microvascular dysfunction [18]. In the heart, both invasive and non-invasive imaging modalities such as echocardiography, computed tomography (CT), cardiovascular magnetic resonance (CMR), positron emission tomography (PET), and coronary angiography—can be useful for detecting microvascular abnormalities [25]. Conversely, in the brain, magnetic resonance imaging (MRI) is the primary modality for identifying microvascular dysfunction and its associated morphological changes [26].

The purpose of this review is to explore the potential common pathophysiological mechanisms linking TS and stroke, with a particular focus on microvascular dysfunction as a shared underlying factor. By examining the role of the heart-brain axis in these conditions and highlighting the critical contribution of advanced imaging techniques, this review aims to clarify how microvascular abnormalities may simultaneously impact both the cardiovascular and cerebrovascular systems. Furthermore, it seeks to provide insights into the clinical implications of these findings and the importance of considering both TS and stroke within the same pathological spectrum.

### Takotsubo syndrome

**Fig. 2:**
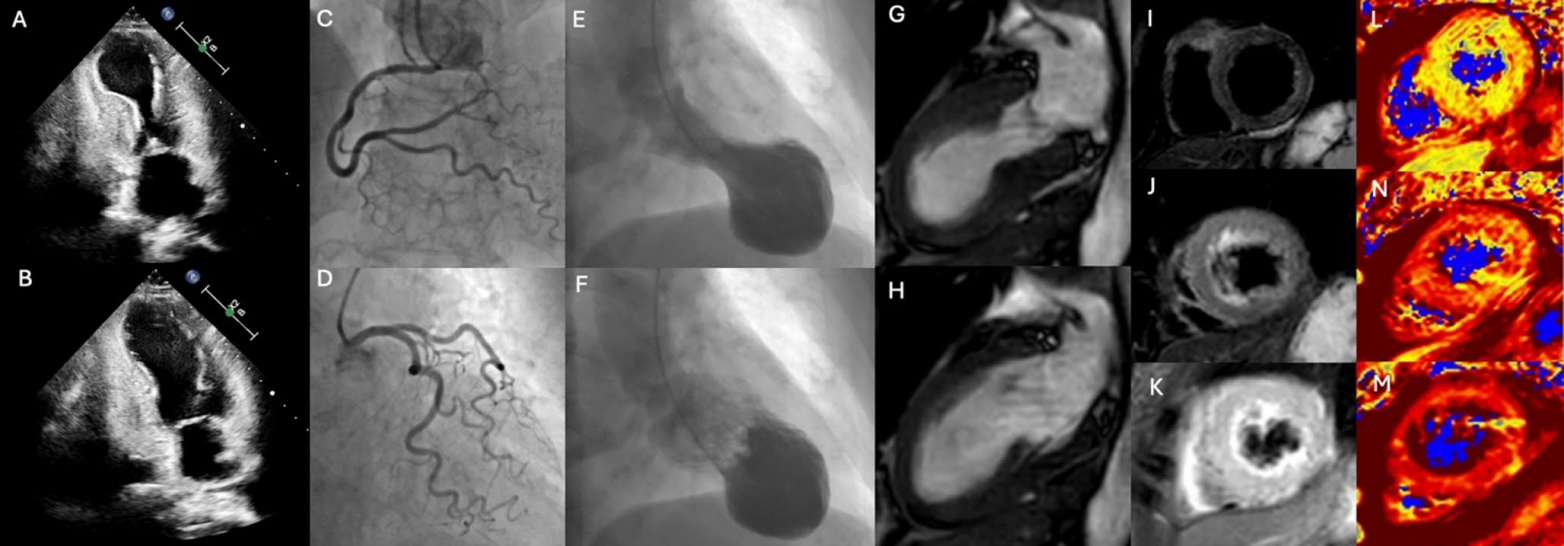
Invasive and non-invasive evaluation in patients with suspected Takotsubo syndrome. Two-dimensional transthoracic echocardiography in apical four-chamber views demonstrates abnormal wall motion in the mid and apical segments (Panel A: end-systolic phase; Panel B: end-diastolic phase).Coronary angiography views of the left and right coronary arteries reveal no significant coronary artery stenosis (Panels C and D).Left ventriculography shows the typical apical ballooning pattern of Takotsubo syndrome (Panels E and F). Cine cardiac magnetic resonance images in the end-systolic (Panel G) and end-diastolic (Panel H) phases illustrate the apical ballooning pattern. T2-weighted short tau inversion recovery and T2 mapping short-axis views at the basal (Panels I, L), mid (Panels J, M), and apical (Panels K, N) levels show diffuse myocardial edema in the segments with wall motion abnormalities

TS is an acute myocardial dysfunction that closely mimics acute myocardial infarction and is often triggered by physical or emotional stress [2–5, 9, 27]. Several diagnostic criteria have been proposed. One of the most widely used is the revised Mayo Clinic Diagnostic Criteria [28], which include transient wall-motion abnormalities in the absence of culprit coronary lesions, myocarditis, or pheochromocytoma. More recently, the International Takotsubo (InterTAK) diagnostic criteria were introduced [4, 5]. According to these guidelines, the presence of obstructive coronary lesions no longer excludes a TS diagnosis, and non-invasive imaging modalities such as CMR are recommended to help differentiate TS from other conditions [4]. CMR, with its unparalleled ability to detect subtle myocardial abnormalities, is considered superior to other imaging techniques for identifying TS [29–33]. Nevertheless, despite the rise of non-invasive techniques, coronary angiography with left ventriculography remains the gold standard for confirming or ruling out TS [4] (Fig. 2**)**.

Although described over 30 years ago, the pathophysiology of TS remains unclear. This contributes to the lack of effective, targeted treatments. TS was initially regarded as a benign, self-limiting condition [6–8, 11, 14, 27]^34^– [42]. However, it is now recognized that TS can lead to significant short- and long-term morbidity and mortality. Identifying its underlying mechanisms is essential for developing targeted therapies for the acute phase and preventing future adverse events. A catecholamine surge remains the most widely accepted theory explaining the pathogenesis of TS.

Stress and severe physical illness or trauma are well-known triggers of TS. This surge activates the locus ceruleus, located in the posterior rostral pons, which subsequently stimulates the hypothalamic-pituitary-adrenal axis [15, 16], leading to norepinephrine release from sympathetic nerve terminals in the myocardium. Despite this understanding, the exact mechanism by which catecholamine excess causes myocardial stunning in TS remains unclear. One prominent hypothesis is microvascular dysfunction, which has been shown—through both invasive and non-invasive methods—to play a central role in the pathophysiology of this syndrome [15, 16]^43^– [49]. A recent in vivo experimental study by Dong et al. has provided compelling insights into the role of microvascular dysfunction in TS [50]. Using a murine model, the authors showed that impairments in the microcirculation can actively precipitate the onset of TS, highlighting microvascular dysfunction as a critical underlying mechanism [50]. Further clinical evidence comes from a prospective study by Ekenback involving 27 female TS patients and 27 sex- and age-matched controls who presented with ischemia and non-obstructive coronary arteries. Using invasive coronary angiography, the authors demonstrated a significantly higher prevalence of microvascular dysfunction in TS patients, with pronounced involvement of the apical myocardial segments [43]. Similarly, Meimoun et al. used Doppler transthoracic echocardiography during the acute phase of TS to assess coronary flow. They found transient microvascular dysfunction, further supporting its pivotal role in the syndrome’s clinical presentation and progression [45].

### Imaging in microvascular dysfunction in the heart

Microvascular dysfunction refers to the impaired function of a complex and diverse network of vessels, typically less than 500 μm in diameter, which plays a key role in regulating myocardial blood flow and facilitating the exchange of oxygen, nutrients, and metabolites. Unlike obstructive coronary artery disease, which affects the larger epicardial vessels, microvascular dysfunction occurs in the smaller vessels that are not easily seen by conventional imaging. The primary consequence of microvascular dysfunction is a mismatch between the myocardial oxygen demand and the microvascular blood supply, which can lead to ischemia, angina, heart failure, and increased cardiovascular risk [51].

Evaluating coronary microcirculation necessitates a functional assessment of the coronary arteries. The 2024 ESC Guidelines for the management of chronic coronary syndromes recommended both invasive and non-invasive methods based on the patient’s profile, the nature of symptoms, comorbidities, and the need for serial evaluations [52].

#### Non-invasive assessment

Echocardiography is one of the most widely available and frequently used imaging techniques in cardiology. For assessing microvascular dysfunction, contrast-enhanced echocardiography and transthoracic Doppler echocardiography are the main approaches [52].

Contrast-enhanced echocardiography uses ultrasound contrast agents, typically microbubbles that remain confined to the vascular space, to assess myocardial perfusion. By administering a continuous infusion of contrast and then using high-energy ultrasound pulses to destroy the microbubbles, echocardiography can measure the rate of microbubble replenishment in the myocardium. This provides an estimate of myocardial blood flow (MBF), based on the average velocity of myocardial microbubbles and the cross-sectional area of the microvasculature [53, 54].

Transthoracic Doppler echocardiography, on the other hand, assesses coronary flow velocity reserve (CFVR), which is the ratio of coronary flow velocity at rest to that during maximal hyperemia, usually induced by a vasodilator such as adenosine [55].

Cardiac CT is primarily used for coronary artery imaging. However, it can also assess microvascular function using CT perfusion (CTP) imaging and CT-derived fractional flow reserve (FFR-CT). CTP imaging can be performed using either static or dynamic scanning during the first pass of contrast through the myocardium, both at rest and under pharmacologic stress [56]. Static CTP provides qualitative perfusion data, while dynamic CTP allows for MBF quantification [57].

Another CT-based technique, FFR-CT, uses computational models derived from coronary CT angiography data to estimate fractional flow reserve non-invasively without stress perfusion imaging [58]. FFR-CT simulates blood flow and pressure within the coronary arteries, offering insights into both epicardial stenosis and microvascular function [59].

One of the most validated methods for assessing microvascular function is PET-CT myocardial perfusion imaging. This technique uses tracers labeled with isotopes that emit positrons and enables the quantification of MBF as well as myocardial flow reserve (MFR). The kinetics of these tracers reflect the extent of MBF increase achievable through maximal coronary vasodilation induced by vasodilators. Since the microvasculature primarily determines vascular resistance, MFR measures the microvasculature’s ability to respond to stimuli, thereby representing small vessel function [25, 51, 59].

Recently, quantitative CMR has emerged as a promising technique for assessing microvascular dysfunction by quantifying MBF through measurements of myocardial signal intensity changes between rest and stress images to evaluate myocardial perfusion reserve [51, 52]. Visual assessment and semi-quantitative methods for perfusion evaluation are commonly employed; however, quantitative MBF analysis using CMR is currently limited to research settings. Visual assessment relies on capturing the first-pass transit of gadolinium in the myocardium, where well-perfused myocardium exhibits a shorter T1 relaxation time, resulting in a brighter appearance, while perfusion deficits manifest as areas of lower signal intensity [25, 26, 59]. Promising results are emerging from first-pass perfusion studies that collect signal intensity data before and after gadolinium administration without necessitating stress imaging [60, 61] (Table 1).

**Table 1:** Advantages and disadvantages of non-invasive imaging modalities in the assessment of microvascular dysfunction

Imaging modality	Pros	Cons
Transthoracic Doppler echocardiography	• Low-risk bedside procedure • Availability • Low-cost • No radiation exposure	• Operator-dependent Limited spatial resolution Limited ability to evaluate non-LAD coronary territories
Contrast enhanced echocardiography	• Low-risk bedside procedure • Availability • Low-cost • No radiation exposure	• Operator-dependent • Limited spatial resolution • Lacking extensive validation
Computed tomography	• High spatial resolution • Anatomic and functional examination in the same examination	• Radiation exposure • Limited availability • Less extensively validated for microvascular dysfunction compared to PET and CMR • Risk of MBF overestimation • Limited by renal function
Cardiovascular magnetic resonance	• No radiation exposure • Excellent spatial resolution • Allows comprehensive tissue characterization • Validated against invasive techniques and PET	• Expensive • Limited availability • Requires frequent breath-holds (difficult for some patients) • Time-consuming​
Positron emission tomography	• Most validated modality for microvascular dysfunction • High sensitivity and temporal resolution • Low radiation exposure (with short-lived tracers) • Strong prognostic value​	• Expensive • Limited availability • Radiation exposure • Requires a cyclotron for some tracers • Time-consuming​

#### Invasive assessment

Invasive coronary function testing is the most comprehensive diagnostic approach for assessing coronary microvascular dysfunction, allowing for the evaluation of both the coronary microcirculation and the presence of epicardial vessel stenosis [62].

Adenosine, a vasodilator acting on smooth muscle cells, is administered to assess coronary flow reserve (CFR), which is calculated as the ratio of hyperemic to resting myocardial blood flow. Both intracoronary Doppler flow wires and temperature sensor-tipped guidewires can be used to measure myocardial blood velocity/flow at rest and after the induction of hyperemia with adenosine [63].

The index of microvascular resistance (IMR) provides a more specific measure of microvascular function. It is calculated as the product of distal coronary pressure and hyperemic mean transit time, using a pressure guidewire and thermodilution techniques. Notably, IMR is independent of epicardial vascular function and serves as a direct marker of the microvascular network [64].

Beyond the assessment of hyperemic measurements, invasive coronary function testing can evaluate macrovascular function and assess for spasm through the administration of intracoronary acetylcholine. Acetylcholine induces vasospasm via cholinergic receptors on vascular smooth muscle cells, leading to nitric oxide release. In cases of endothelial dysfunction, this mechanism is impaired, as the endothelium cannot release sufficient nitric oxide to counteract the muscarinic receptor stimulation, resulting in vasoconstriction [63, 64] (Table 2).

**Table 2:** Advantages and disadvantages of invasive imaging modalities in the assessment of microvascular dysfunction

Imaging modality	Pros	Cons
Coronary Angiography	• Simple procedure • No additional contrast or radiation required​	• Semi-quantitative assessment • Limited sensitivity for detecting microvascular dysfunction​
Intracoronary Thermodilution	• High success rate of measurements • Specific to microcirculation (not affected by resting hemodynamics) • Validated against clinical outcomes​	• High intra- and inter-observer variability • Varying cut-off values across clinical studies​
Intracoronary Doppler Flow	• Provides direct measurement of coronary peak flow velocity • Widely available and low cost​	• Technically complex • Controversial cut-off values
Intracoronary Vasoreactivity Testing	• Simple method • Can assess both epicardial and microvascular spasm • Does not require additional equipment	• Requires additional contrast and radiation • Risk of arrhythmias during testing​

### Imaging in microvascular dysfunction in the brain

MRI can detect small cerebral vessel impairment by providing high-resolution images of the brain, revealing structural changes and abnormalities associated with small vessel disease. Recently, the international working group on the Standards for Reporting Vascular Changes on Neuroimaging (STRIVE) recommended standardized classifications for describing neuroimaging features [65].

**Fig. 3:**
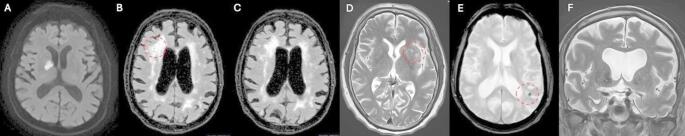
Examples of small cerebral vessel impairment using MRI according to the Standards for Reporting Vascular Changes on Neuroimaging (STRIVE) recommended standardized classifications, including small subcortical infarcts (panel a), lacunes of presumed vascular origin (panel b), white matter hyperintensities of presumed vascular origin (panel c), perivascular spaces (panel d), cerebral microbleeds (panel e), and brain atrophy(panel f)

These included recent small subcortical infarcts, lacunes of presumed vascular origin, white matter hyperintensities of presumed vascular origin, dilated perivascular spaces, cerebral microbleeds, and brain atrophy [60] (Fig. 3). Several studies have proposed scoring systems that combine these markers into a total cerebral small vessel disease score, representing the overall disease burden in the brain [66–68]. White matter is particularly vulnerable to the effects of microvascular dysfunction because it receives significantly less blood perfusion compared to gray matter [69].

White matter consists of bundles of axonal fibers that facilitate the rapid and efficient transmission of signals between different brain regions. Given its lower perfusion, the white matter is more susceptible to ischemic injury, making it highly sensitive to even subtle disruptions in microvascular blood flow. Impaired perfusion can lead to structural damage or degeneration of white matter tracts, which may reduce the speed and integrity of neural signal transmission. This, in turn, can alter brain connectivity [70].

**Fig. 4:**
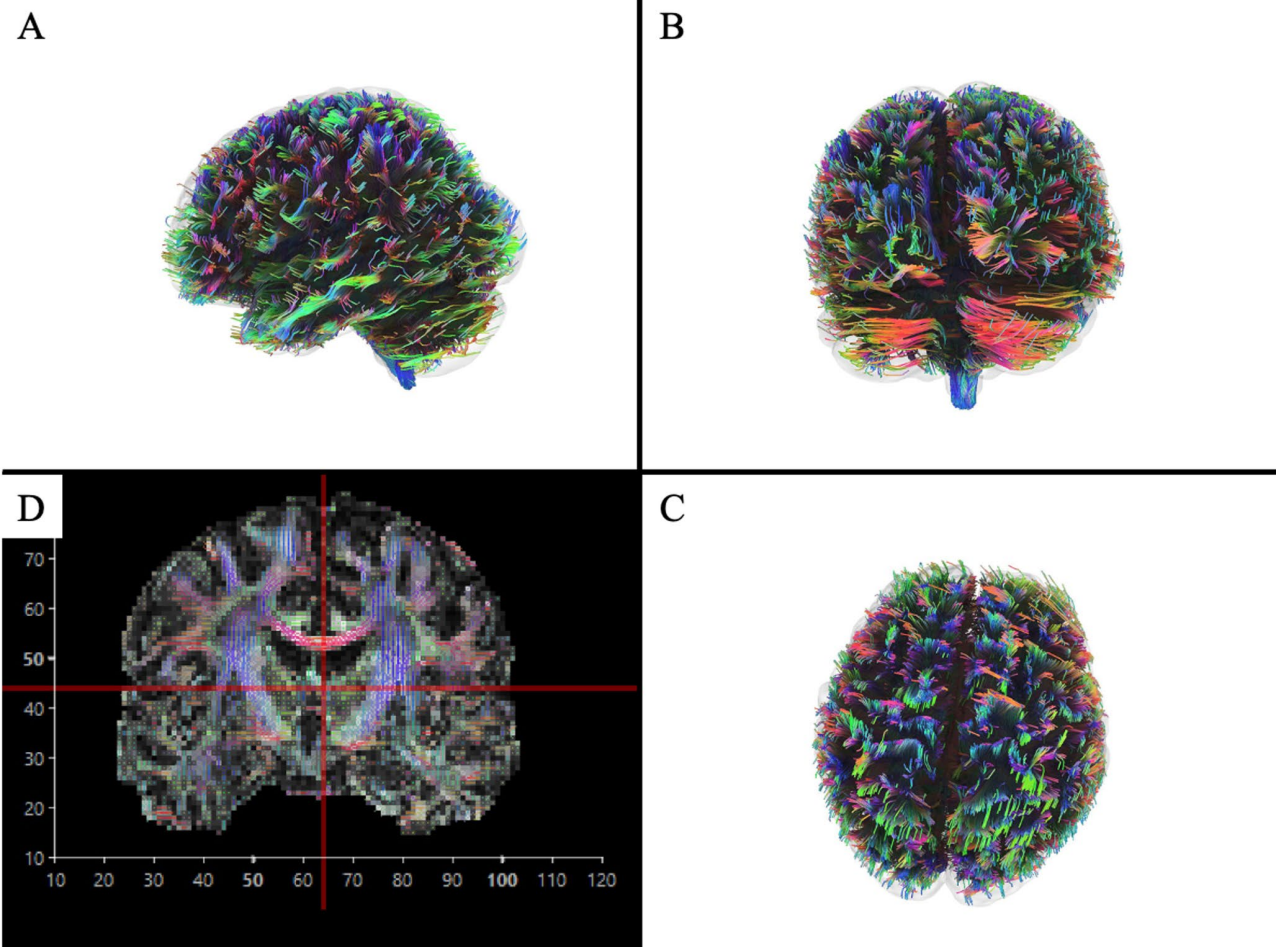
Example of DTI imaging in a patient with suspected Takotsubo syndrome

Diffusion tensor imaging (DTI) is a valuable tool to assess white matter integrity. It measures the magnitude and direction of water diffusion within a tissue, providing insights into white matter integrity and microvascular dysfunction [71, 72] (Fig. 4).

Several key scalar measures are derived, including: (1) Mean diffusivity (MD) reflects the average rate of water diffusion in all directions, providing a general sense of tissue health; in particular, MD tends to increase in vasogenic edema and encephalomalacia and decrease in cytotoxic edema [73] (2) Fractional anisotropy (FA) measures the degree of directionality or coherence of water movement, with higher values indicating diffusion primarily in a principal direction in parallel to intact nerve fibers. Further, it has been demonstrated that FA tends to decrease in cases of demyelination, interstitial edema and axonal loss [73, 74]. (3) Axial diffusivity (AD) quantifies water diffusion along the length of the axonal tracts, giving insight into the integrity of the axons themselves; in particular, this value is nonspecifically associated with axonal density, and it has been demonstrated that it tends to reduce in those conditions characterized by axonal loss [72, 73, 75]. (4) Radial diffusivity (RD) captures water diffusion perpendicular to the axonal tracts, which is often associated with damage to the myelin sheath surrounding the axons and/or axonal loss and increases in demyelinating processes [72, 73].

Other advanced MRI techniques, such as dynamic contrast-enhanced MRI (DCE-MRI), quantitative Susceptibility Mapping and assessments of cerebrovascular reactivity, have been proposed to provide new pathophysiological insights into cerebral small vessel disease [76–79].

DCE-MRI evaluates increased blood-brain barrier permeability resulting from endothelial dysfunction. By measuring the rate at which the contrast agent moves into the extravascular space per unit of tissue volume and its concentration, DCE-MRI offers a quantitative assessment of blood-brain barrier permeability [76]. Conversely, quantitative susceptibility mapping (QSM) measures the magnetizability of a material in response to an applied magnetic field. This technique provides substance-specific values and can reveal heterogeneity in the composition of white matter hyperintensities based on their location, highlighting potential differences in pathophysiological mechanisms across brain regions [76, 78, 79].

Another non-invasive imaging modality used to identify vascular dysfunction in patients with cerebral small vessel disease is cerebrovascular reactivity (CVR). This technique evaluates the ability of cerebral blood vessels to dilate in response to increased brain energy demand by measuring changes in blood oxygen level-dependent (BOLD) signals. Various methods are used to provoke vasodilation, ranging from pharmacological approaches, such as administering acetazolamide, to inhaling carbon dioxide-enriched air [76, 77].

### Brain-heart axis in TS: shared signaling

Microvascular dysfunction refers to to impaired regulation of blood flow in the small vessels. In the coronary circulation, this dysfunction is closely linked to cerebral small vessel disease and reduced cerebral blood flow, suggesting an intricate relationship between cardiac and cerebral microvascular health. Evidence from the Cerebral-Coronary Connection (C3) study, which included 73% women, highlights the association between coronary microvascular dysfunction and the burden of white matter hyperintensities on brain MRI [80]. Patients with coronary microvascular dysfunction also showed reduced FA and MD values on brain DTI, potentially indicating axonal degeneration and demyelination, serves as an early marker of cerebral small vessel disease [80].

Similar findings were reported in a pilot study by Mazini et al., which examined microvascular function in 370 patients using quantitative ^82^Rb cardiac PET/CT and brain MRI. The study showed that patients with cardiac small vessel disease exhibited a greater extent of frontal white matter atrophy, ventricular dilation, and a higher volume of deep gray matter lacunae compared to those with normal cardiac perfusion imaging [81].

The multisystemic nature of small vessel diseases was also highlighted in the systematic review by Barry et al. [82]. TS is characterized by heightened sympathetic nerve activity, particularly in key brain regions. The limbic system, neocortex, spinal cord, reticular formation, and brainstem are critical components of the neuroanatomic pathways involved in the stress response. Altered functional connectivity patterns in TS patients were recently demonstrated by Templin et al. using brain functional magnetic resonance imaging. The authors reported that TS patients exhibited reduced resting-state functional connectivity in brain regions associated with parasympathetic and sympathetic subnetworks [83]. Stress-induced activation of the sympathetic nervous system triggers a sudden surge in plasma catecholamines [84].

The link between catecholamine elevation and endothelial dysfunction was recently demonstrated in both in vivo animal and in vitro studies. These studies revealed that the β2-adrenoceptor-Giα signaling pathway and the α1 receptor/Gq/PKC signaling pathway play key roles in the vasoconstrictor response induced by endothelial oxidative stress, accompanied by increased reactive oxygen species signaling [20, 85].

This association between catecholamine surges and endothelial dysfunction has also been observed in patients with pheochromocytoma, where excessive catecholamine secretion impairs vasodilatory mechanisms, leading to vasoconstriction. Notably, endothelial function is promptly restored following adrenalectomy [21].Importantly, the vasoconstrictive effects of catecholamine surges in pheochromocytoma are not confined to the heart but can result in multisystemic endothelial dysfunction, as evidenced by cases of reversible cerebral ischemia in these patients [19, 86, 87].

As part of the brain-heart axis, the blood-brain barrier responds to hypoxic events triggered by cardiac impairment, and this relationship is reciprocal. In vivo studies have shown that systemic administration of epinephrine can disrupt the integrity of the blood-brain barrier [88], potentially initiating or worsening cerebral microvascular disease [89–91].

Collectively, previous reports suggest that acute surges in catecholamines may induce widespread endothelial dysfunction, contributing to the transient myocardial impairment characteristics of TS [86, 87, 92].

This catecholamine surge may also compromise the integrity of the blood-brain barrier, as elevated catecholamine levels can disrupt endothelial cell function and increase blood-brain barrier permeability. Such disruption could enhance susceptibility to cerebrovascular complications, including an increased risk of stroke, highlighting a potential connection between TS-related myocardial and cerebrovascular vulnerabilities [93].

## Clinical implications

A thorough understanding of TS pathophysiology and its relationship with cerebral small vessel disease and consequent stroke could aid in developing more targeted therapies for managing TS patients. Assessing microvascular dysfunction through both invasive and non-invasive methods to identify an impaired systemic microvascular network in TS could support clinicians in implementing tailored management strategies. From a prognostic standpoint, microvascular dysfunction in TS patients is linked to adverse cardiovascular outcomes both during hospitalization and over the long term [6, 7, 94, 95]. Early recognition of this dysfunction may enable more personalized management strategies, with closer monitoring and potential interventions to improve microvascular function. Moreover, future clinical studies are needed to confirm the triggering role of myocardial microvascular dysfunction in TS, investigate its temporal relationship with stroke, and evaluate its long-term impact on outcomes and overall survival.

## Future perspectives

A deeper understanding of the heart–brain axis—particularly the bidirectional relationship between TS and stroke—is emerging as both a challenge and an opportunity in cardiovascular and neurovascular medicine. Advances in omics technologies—including genomics, transcriptomics, proteomics, and metabolomics—are creating new possibilities for uncovering the molecular mechanisms underlying disease susceptibility, onset, and recurrence. For instance, genetic variants involved in stress-response pathways, circulating biomarkers of neurohormonal dysregulation, and metabolomic profiles associated with endothelial or microvascular dysfunction may help identify individuals at increased risk and support more personalized therapeutic strategies [96–101]. At the same time, artificial intelligence is poised to revolutionize this field by integrating complex, multimodal datasets, including clinical information, imaging findings, and high-dimensional omics data [102–105]. This integration holds great promise for developing predictive models, enabling earlier detection, and guiding individualized treatment approaches for patients affected by or at risk of heart–brain axis disorders.

## Conclusion

The microvascular dysfunction in TS patients is not organ-specific but part of a systemic disorder affecting the microcirculation of multiple organs, including the heart and brain. Further studies are needed to determine whether sympathetic nervous system-induced microcirculatory dysfunction may contribute to the increased prevalence of stroke in TS patients.

## Data Availability

No datasets were generated or analysed during the current study.
